# What were the historical reasons for the resistance to recognizing airborne transmission during the COVID‐19 pandemic?

**DOI:** 10.1111/ina.13070

**Published:** 2022-08-21

**Authors:** Jose L. Jimenez, Linsey C. Marr, Katherine Randall, Edward Thomas Ewing, Zeynep Tufekci, Trish Greenhalgh, Raymond Tellier, Julian W. Tang, Yuguo Li, Lidia Morawska, Jonathan Mesiano‐Crookston, David Fisman, Orla Hegarty, Stephanie J. Dancer, Philomena M. Bluyssen, Giorgio Buonanno, Marcel G. L. C. Loomans, William P. Bahnfleth, Maosheng Yao, Chandra Sekhar, Pawel Wargocki, Arsen K. Melikov, Kimberly A. Prather

**Affiliations:** ^1^ Department of Chemistry and Cooperative Institute for Research in Environmental Sciences University of Colorado Boulder Colorado USA; ^2^ Department of Civil and Environmental Engineering Virginia Tech Blacksburg Virginia USA; ^3^ Department of English Virginia Tech Blacksburg Virginia USA; ^4^ Department of History Virginia Tech Blacksburg Virginia USA; ^5^ School of Journalism Columbia University New York New York USA; ^6^ Department of Primary Care Health Sciences Medical Sciences Division University of Oxford Oxford UK; ^7^ Department of Medicine McGill University Montreal Québec Canada; ^8^ Department of Respiratory Sciences University of Leicester Leicester UK; ^9^ Department of Mechanical Engineering University of Hong Kong Hong Kong China; ^10^ International Laboratory for Air Quality and Heath Queensland University of Technology Brisbane Queensland Australia; ^11^ Goldman Hine LLP Toronto Ontario Canada; ^12^ Dalla Lana School of Public Health University of Toronto Toronto Ontario Canada; ^13^ School of Architecture, Planning & Environmental Policy University College Dublin Dublin Ireland; ^14^ Department of Microbiology Hairmyres Hospital, Glasgow, and Edinburgh Napier University Glasgow UK; ^15^ Faculty of Architecture and the Built Environment Delft University of Technology Delft The Netherlands; ^16^ Department of Civil and Mechanical Engineering University of Cassino and Southern Lazio Cassino Italy; ^17^ Department of the Built Environment Eindhoven University of Technology (TU/e) Eindhoven The Netherlands; ^18^ Department of Architectural Engineering The Pennsylvania State University University Park Pennsylvania USA; ^19^ College of Environmental Sciences and Engineering Peking University Beijing China; ^20^ Department of the Built Environment National University of Singapore Singapore Singapore; ^21^ Department of Civil Engineering Technical University of Denmark Lyngby Denmark; ^22^ Scripps Institution of Oceanography University of California San Diego La Jolla California USA

**Keywords:** airborne transmission, disease transmission, droplet transmission, history

## Abstract

The question of whether SARS‐CoV‐2 is mainly transmitted by droplets or aerosols has been highly controversial. We sought to explain this controversy through a historical analysis of transmission research in other diseases. For most of human history, the dominant paradigm was that many diseases were carried by the air, often over long distances and in a phantasmagorical way. This miasmatic paradigm was challenged in the mid to late 19th century with the rise of germ theory, and as diseases such as cholera, puerperal fever, and malaria were found to actually transmit in other ways. Motivated by his views on the importance of contact/droplet infection, and the resistance he encountered from the remaining influence of miasma theory, prominent public health official Charles Chapin in 1910 helped initiate a successful paradigm shift, deeming airborne transmission most unlikely. This new paradigm became dominant. However, the lack of understanding of aerosols led to systematic errors in the interpretation of research evidence on transmission pathways. For the next five decades, airborne transmission was considered of negligible or minor importance for all major respiratory diseases, until a demonstration of airborne transmission of tuberculosis (which had been mistakenly thought to be transmitted by droplets) in 1962. The contact/droplet paradigm remained dominant, and only a few diseases were widely accepted as airborne before COVID‐19: those that were clearly transmitted to people not in the same room. The acceleration of interdisciplinary research inspired by the COVID‐19 pandemic has shown that airborne transmission is a major mode of transmission for this disease, and is likely to be significant for many respiratory infectious diseases.

**Practical Implications**

Since the early 20th century, there has been resistance to accept that diseases transmit through the air, which was particularly damaging during the COVID‐19 pandemic. A key reason for this resistance lies in the history of the scientific understanding of disease transmission: Transmission through the air was thought dominant during most of human history, but the pendulum swung too far in the early 20th century. For decades, no important disease was thought to be airborne. By clarifying this history and the errors rooted in it that still persist, we hope to facilitate progress in this field in the future.

## 1 INTRODUCTION

The COVID‐19 pandemic motivated an intense debate over the modes of transmission of the SARS‐CoV‐2 virus, involving mainly three modes: First, impact of “sprayborne” droplets on eyes, nostrils, or mouth, that otherwise fall to the ground close to the infected person. Second, by touch, either by direct contact with an infected person, or indirectly by contact with a contaminated surface (“fomite”) followed by self‐inoculation by touching the interior of the eyes, nose, or mouth. Third, upon inhalation of aerosols, some of which can remain suspended in the air for hours (“airborne transmission”).^1^, ^2^

Public health organizations including the World Health Organization (WHO) initially declared the virus to be transmitted in large droplets that fell to the ground close to the infected person, as well as by touching contaminated surfaces. The WHO emphatically declared on March 28, 2020, that SARS‐CoV‐2 was not airborne (except in the case of very specific “aerosol‐generating medical procedures”) and that it was “misinformation” to say otherwise.^3^ This advice conflicted with that of many scientists who stated that airborne transmission was likely to be a significant contributor. e.g. Ref.^4^, ^5^, ^6^, ^7^, ^8^, ^9^ Over time, the WHO gradually softened this stance: first, conceding that airborne transmission was possible but unlikely;^10^ then, without explanation, promoting the role of ventilation in November 2020 to control spread of the virus (which is only useful for controlling airborne pathogens);^11^ then declaring on April 30, 2021, that transmission of SARS‐CoV‐2 through aerosols is important (while not using the word “airborne”).^12^ Although a high‐ranking WHO official admitted in a press interview around that time that “the reason we're promoting ventilation is that this virus can be airborne,” they also stated that they avoided using the word “airborne.”^13^ Finally in December 2021, WHO updated one page in its website to clearly state that short‐ and long‐range airborne transmission are important, while also making clear that “aerosol transmission” and “airborne transmission” are synonyms.^14^ However, other than that web page, the description of the virus as “airborne” continues to be almost completely absent from public WHO communications as of March 2022.

The Centers for Disease Control and Prevention (CDC) in the United States followed a parallel path: first, stating the importance of droplet transmission; then, in September 2020, briefly posting on its website an acceptance of airborne transmission that was taken down three days later;^15^ and finally, on May 7, 2021, acknowledging that aerosol inhalation is important for transmission.^16^ However, CDC frequently used the term “respiratory droplet,” generally associated with large droplets that fall to the ground quickly,^17^ to refer to aerosols,^18^ creating substantial confusion.^19^ Neither organization highlighted the changes in press conferences or major communication campaigns.^20^ By the time these limited admissions were made by both organizations, the evidence for airborne transmission had accumulated, and many scientists and medical doctors were stating that airborne transmission was not just a possible mode of transmission, but likely the *predominant* mode.^21^ In August 2021, the CDC stated that transmissibility of the delta SARS‐CoV‐2 variant approached that of chickenpox, an extremely transmissible airborne virus.^22^ The omicron variant that emerged in late 2021 appeared to be a remarkably fast spreading virus, exhibiting a high reproductive number and a short serial interval.^23^

The very slow and haphazard acceptance of the evidence of airborne transmission of SARS‐CoV‐2 by major public health organizations contributed to a suboptimal control of the pandemic, whereas the benefits of protection measures against aerosol transmission are becoming well established.^24^, ^25^, ^26^ Quicker acceptance of this evidence would have encouraged guidelines that distinguished rules for indoors and outdoors, greater focus on outdoor activities, earlier recommendation for masks, more and earlier emphasis on better mask fit and filter, as well as rules for mask‐wearing indoors even when social distancing could be maintained, ventilation, and filtration. Earlier acceptance would have allowed greater emphasis on these measures, and reduced the excessive time and money spent on measures like surface disinfection and lateral plexiglass barriers, which are rather ineffective for airborne transmission and, in the case of the latter, may even be counterproductive.^29^, ^30^

Why were these organizations so slow, and why was there so much resistance to change? A previous paper considered the issue of scientific capital (vested interests) from a sociological perspective.^31^ Avoiding costs associated with measures needed to control airborne transmission, such as better personal protective equipment (PPE) for healthcare workers^32^ and improved ventilation^33^ may have played a role. Others have explained the delay in terms of perception of hazards associated with N95 respirators^32^ that have, however, been disputed^34^ or because of poor management of emergency stockpiles leading to shortages early in the pandemic. e.g. Ref.^35^

An additional explanation not offered by those publications, but which is entirely consistent with their findings, is that the hesitancy to consider or adopt the idea of airborne transmission of pathogens was, in part, due to a conceptual error that was introduced over a century ago and became ingrained in the public health and infection prevention fields: a dogma that transmission of respiratory diseases is caused by large droplets, and thus, droplet mitigation efforts would be good enough. These institutions also displayed a reluctance to adjust even in the face of evidence, in line with sociological and epistemological theories of how people who control institutions can resist change, especially if it seems threatening to their own position; how groupthink can operate, especially when people are defensive in the face of outsider challenge; and how scientific evolution can happen through paradigm shifts, even as the defenders of the old paradigm resist accepting that an alternative theory has better support from the available evidence.^36^, ^37^, ^38^ Thus, to understand the persistence of this error, we sought to explore its history, and of airborne disease transmission more generally, and highlight the key trends that led to droplet theory becoming predominant.

## 2 METHOD

Focusing mainly on infections acquired through the airways (such as tuberculosis, smallpox, measles, and influenza) and others that were thought historically to transmit through the air (such as malaria and cholera), we collected historical theories and models of disease transmission from the ancient Greeks to the present day. Beginning with sources on this topic that were known to the authors, we used backward tracking (pursuing references of those sources) and forward tracking (tracking the source forward in Google Scholar to see which subsequent sources cited it). We also used literature searches in PubMed, Google Scholar, and Web of Science, as well as consultation with experts to identify other key papers on the same topics. Only literature in the English language was systematically searched, although some references in other languages were reviewed and a few of them are cited. We searched for the origins of the resistance to recognizing airborne transmission during the COVID‐19 pandemic, especially by leading public health institutions like WHO, which appeared to be rooted in Western scientific tradition. We acknowledge that other nations had their own views about respiratory disease transmission throughout history, but we do not explore those in this article. We used hermeneutic methods to produce a narrative synthesis of this literature, building a progressively richer picture of how the transmission of particular diseases had originally been conceptualized and what empirical evidence had led scientists to revise the model of transmission. To refine our interpretation, we explicitly sought disconfirming studies (e.g., we looked for ones that challenged prevailing models and assumptions).

## 3 FINDINGS

### 3.1 Disease transmission throughout most of human history: miasmas and infective air

Humanity has been wrestling with the mystery of disease transmission for over two millennia. After all, figuring out how contagious diseases spread is difficult. When a person falls ill, we need to consider which of the many things they did (and in particular, to which infectious agents they were *exposed to*) led to infection. As we will see, time and again, this difficulty made it hard to tell exactly how people became sick, and led to incorrect theories of transmission becoming entrenched, and it was then very difficult to dislodge them despite strong evidence in support of a rival theory. Transmission through the air is especially difficult to precisely pinpoint, given that the infectious particles are invisible and air moves with fewer restrictions, compared to, for example, transmission through water, food, hands, or mosquitoes.

However, it should be noted that establishing transmission methods was difficult for other mechanisms as well, both for scientific and sociological reasons. For example, the (then‐unknown) incubation period muddied multiple experiments and systematic observations trying to connect mosquitos and yellow fever.^39^ Similarly, the co‐occurrence of poverty and malnutrition with filthy air and unclean water helped confuse the fact of water‐borne transmission of cholera.^40^ Without microscopes and a germ theory of disease, it is difficult distinguish among various plausible pathways. Plus, both John Snow and Ignaz Semmelweis faced stiff resistance from the scientific establishment of their day, with some similarities to the resistance to accepting airborne transmission from the current establishment.^40^, ^41^

Hippocratic writings in ancient Greece first proposed that diseases were caused by imbalance of humors in the body, which could be triggered by a “miasma” transmitted through the air: “Whenever many men are attacked by one disease at the same time, the cause should be assigned to that which is most common, and which we all use most. This it is which we breathe in.”^42^ Postulated imbalances between humors also gave rise to a theory of personality types, for example “melancholia” was ascribed to an excess of black bile (“melaina chole”). Throughout much of subsequent human history, the belief persisted that diseases were transmitted through the air. Because the actual causative agents of airborne diseases remained a mystery for centuries, explanations were given in general terms such as “miasmas,” or “bad air”^43^ as illustrated by the etymological root of the term malaria (from “mala aria,” medieval Italian for “bad air”). Some origin theories were more specific than others. For example, Roman scholar Marcus Terentius Varro (116–27 BCE) wrote that swamps were a particular breeding ground for minute creatures that “float in the air and enter the body through the mouth and nose and there cause serious diseases.”^43^ Based on these considerations, it became a policy of the Roman Empire to drain swamps, which removed breeding grounds for mosquitoes and reduced the incidence of malaria, an example of a mistaken theory nevertheless giving good results. Regardless of whether transmitted or triggered by bad humors or minute creatures, airborne infections were generally not viewed as contagious and transmitted from human to human. Rather, infection was believed to simply flow through the air and strike people down.

Persian physician Ibn Sina (Avicenna) in his *Canon of Medicine* in 1025 summarized the classical Greco‐Roman miasma theory, but also blended with it the idea that people could transmit disease to others by breath.^44^ However, the theory of person‐to‐person transmission of disease via infection was not clearly formulated until Italian physician Girolamo Fracastoro (Fracastorius) (1478–1553) proposed it in 1546.^45^ This idea was built upon a “seeds” theory by Galen of Pergamon, a prolific Greek physician and writer (162 to 203 CE).^46^ Galen's seeds theory had not caught on, probably because he expressed it somewhat tentatively, and his more extensive writings continuing Hippocratic humoral theory overshadowed it.^47^ Interestingly, Fracastoro's book proposed that the seeds of disease‐causing contagion, or “seminaria” as he called them, transmitted through three modes: direct, indirect, and at a distance. Contagion at a distance was, he suggested, the strongest, stronger even than direct contagion. From his writings, these seeds could be interpreted as chemicals rather than living organisms.

In 1590, less than half a century after Fracastoro's writings, spectacle‐makers Hans and Zacharias Janssen invented the microscope. This invention was quickly used by other scientists to discover microorganisms.^48^ Microscopic fungi were discovered by Robert Hooke in 1665, who published his famous *Micrographica* in 1667.^49^ Bacteria were discovered by Antoni van Leeuwenhoek in 1676. These discoveries were a notable step forward; they demonstrated the ubiquity of tiny living creatures too small to be seen by the naked eye and yet potentially capable of causing diseases. What ensued after Fracastoro's pronouncement, however, was a centuries‐long debate between “miasmatists,” who held fast to the idea that diseases floated through the air over distances, and “contagionists,” who accepted person‐to‐person spread of disease.^50^

Because, as stated earlier, it was very difficult to determine how, why, and from where someone became infected, the debate failed to reach a resolution. Observations of outbreaks would sometimes note that quarantine did not work, suggesting the miasmatists were correct. On the contrary, people were not always struck down from afar, suggesting that perhaps it was contagion causing the illness. A middle ground was eventually proposed, called “contingent contagionism,” which was a way of modulating the use of the term “contagious disease” for a specific infection. Contingent contagionism could hold, for example, that malaria, or cholera might be contagious in an impure atmosphere, but might not be contagious in a healthy atmosphere.^51^ This idea, derived from observation, therefore captured some grains of truth, since for example airborne diseases are much more contagious in indoors locations with poor ventilation.^21^

Florence Nightingale (1820–1910) like most Victorians was raised to believe that diseases were caused by ‘miasma’ or foul air. In her *Notes on Hospitals, she wrote:* “What does ‘contagion’ mean? It implies the communication of disease from person to person by *contact*. [ …] There is no end to the absurdities connected with this doctrine. Suffice it to say that […] there is no proof […] that there is any such thing as ‘contagion’. Infection acts through the air. Poison the air breathed by individuals, and there is infection.”^52^ However, she collaborated with contingent contagionists on sanitary measures. She reduced infection rates with hygiene, ventilation, increasing the distance between beds in hospitals, and creating an “isolation ward” for tuberculosis patients. She encountered significant resistance from her family over her chosen profession, and from military superiors for implementing basic hygiene practices during the Crimean War. Later on in her career, the British government finally accepted her sanitary and other reforms after years of lobbying.^52^, ^53^, ^54^

### 3.2 Snow, Semmelweis, and the public health establishment

In 1854, a cholera epidemic struck London. The public health establishment believed it to be caused by a miasma. English sanitary reformers such as Sir Edwin Chadwick, who initiated many modern public health practices,^55^ found miasma theory appealing, as it appeared to explain the prevalence of diseases in the undrained, filthy, and foul‐smelling areas where the poor lived, and helped justify their efforts to address those conditions.^56^

John Snow, a wealthy doctor but an outsider to public health, whose work in anesthesia made him familiar with the behavior of gasses, realized that the spread was not consistent with what would be expected for a gas. He noticed how cases had clustered in a specific London borough and persuaded the local council to remove the handle of the Broad street water pump, which halted the epidemic.^57^ However, by the time he did this, the epidemic was already in decline and so the Board of Health in the end refused to accept contaminated water as the explanation, issuing a report stating “[w]e see no reason to adopt this belief [that cholera was water‐borne],” and dismissing Snow's conclusions as mere “suggestions.”^40^ Snow died before his discovery was accepted in 1866.^40^ The Sanitarians had strong incentives for rejecting water as the source of cholera. To remove the sources of the miasma (filth), they had spearheaded the effort to build sewers that dumped raw sewage into the Thames, the source of much of London's drinking water, thus effectively helping the spread of cholera. They had much to lose by admitting cholera transmitted through water, including their prestige.

Ignaz Semmelweis was another pioneer of disease transmission who was also initially ignored as having proposed things too radical for the establishment of the time to accept. Working in Vienna in 1847, he showed that handwashing greatly reduced deaths by childbed fever in a maternity clinic.^41^ However, his ideas conflicted with established medical and scientific beliefs that still described diseases as due to an imbalance of humors triggered by a miasma in the air.^58^ Thus, the idea that washing hands would reduce disease made no sense to the medical doctors at the time. Not helping matters, his colleagues resented not only his brash style but also the implication that they were hurting their patients by not handwashing, and he was largely ignored, rejected, or ridiculed. Although his data were compelling, he was dismissed from his hospital and harassed by the Vienna medical community so much that eventually he was forced to move to Budapest. After some years there, he broke down, was interned and beaten by the guards, and ultimately died from an infected wound. As with Snow, Semmelweis never saw the fruits of his work, as the importance of handwashing to reduce infection was only accepted by the medical community more than 20 years after his death. In an ironic turn, Semmelweis' name lives on not only for his advances of hand sanitation, but also in the term “Semmelweis reflex,” which has been coined to describe the reflex‐like tendency to reject new knowledge or evidence when it contradicts established beliefs, norms, or paradigms.^59^, ^60^

### 3.3 Second half of 19th century: germ theory

In the second half of the 19th century, Pasteur and Koch offered evidence to support their germ theory of disease. In 1861, Pasteur conducted experiments disproving the spontaneous generation and proving there are viable microorganisms in the air.^61^ However, germ theory was not accepted overnight, and it too encountered much resistance. For example, experiments by others in which water containing organic matter was boiled in a vessel, but microorganisms still appeared (later shown to be due to an imperfect seal or insufficient boiling time) created significant controversy at the time.^62^ But by the late 1880s, miasma theory was waning in popularity, and in 1888, the Institut Pasteur was created in Paris, reflecting the ascendancy of germ theory. Florence Nightingale did accept the new ideas of germ theory, in fact before many physicians did. For example, in 1882, she wrote “Always have chlorinated soda for nurses to wash their hands, especially after dressing or handling a suspicious case. It may destroy germs at the expense of the cuticle, but if it takes off the cuticle, it must be bad for the germs.”^63^ Initial results on some plant pathogens in the 1890s^64^, ^65^ and the identification of the first bacteriophage in 1917 paved the way for the rrecognition of viruses.^66^, ^67^ A “golden era” followed, with the identification of the actual microorganisms that cause many infectious diseases.

The discovery and identification of the organisms causing different diseases did not, however, eliminate the great difficulty in conclusively determining the mode by which they transferred from one person to another. For example, French physician Charles Laveran identified the pathogen responsible for malaria in 1880, but the manner of transmission was still thought to be through the air. American physician Albert Freeman Africanus King proposed that malaria was transmitted by mosquitoes, but encountered general skepticism. In 1883, he presented a list of 19 facts that supported mosquitoes as the vector of malaria transmission. King had correctly identified the co‐occurrence of mosquitoes and malaria, but mistakenly posited that transmission was through their eggs, not bites, in yet another example of the complexities of causal inference for transmission of diseases.^68^ However, the theory was not accepted until 1898 when British surgeon Ronald Ross provided definitive evidence, confirming the presence of the malarial parasites in mosquitoes, and demonstrating transmission of bird malaria by mosquitoes.^43^

In the 1890s, Carl Flügge in Germany set out to disprove the then‐dominant transmission theory for tuberculosis, one of the major infectious diseases of the time. Most experts believed that tuberculosis was transmitted when dust of dried sputum that had landed on floors, blankets, bowls, and other contaminated objects was dispersed into the air. In contrast, Flügge thought that it was not the *dried* secretions from the sick that caused infection, but rather fresh secretions that people were exposed to in air before they reached the ground.^69^ Some contemporaries of Flügge such as Cornet argued that tuberculosis was transmitted only through large droplets, which were easily visible to the naked eye.^70^ Cornet was very concerned about the social implications of infected air, stating “If not only the sputum, but the exhaled air […] contains bacteria, then we have no choice but to put our feet on our laps and be resigned, his fate reaches us too with an infected breath. Terrible then is the fate of those suffering […] like the lepers of earlier centuries have to be expelled from human society.”

However, although the term “Flügge's droplets” has been used to describe only those large particles that fell to the ground quickly near the infected person and that were assumed to dominate transmission, e.g. Ref.^71^ that does not accurately capture Flügge's results. Rather, Flügge and collaborators used the term “droplet” to refer to fresh particles of all sizes, including aerosols for which the researchers waited 5 h to settle from the air on their collection plates.^69^

Investigation of airborne infection continued. In 1905, microbiologist M.H. Gordon was commissioned to study the atmospheric hygiene of the UK House of Commons after an epidemic of influenza among members. He famously performed the following experiment: after gargling with a broth culture of *Serratia marcescens* (formerly known as *Monas prodigiosus*, *Bacillus prodigiosus,* and other names; environmental strains produce a bright red pigment making colonies unmistakable, and the bacterium has often been used as a biological marker), he loudly recited passages from Shakespeare in an empty House to an audience of agar plates, in order to investigate the spatial reach of pathogen‐containing aerosols and droplets. Although growth of colonies was more numerous on plates near the speaker, cultures were apparent on some plates over 21 m away.^72^, ^73^ However, progress was hampered by the limitations of the experimental techniques available at the time.

### 3.4 Charles Chapin, contact infection, and the key errors

The critical point in this history of the understanding of airborne disease transmission is the work of prominent American epidemiologist, Charles V. Chapin. Chapin worked only a couple of decades after the germ theory was accepted, during a period of intense research on pathogen transmission. It was a fluid time, following a major paradigm shift, in which it was easier to change the dominant scientific discourse than during normal times.^38^ He summarized the evidence of transmission of different diseases in his 1910 seminal book, “The Sources and Modes of Infection.”^74^ Based on his own success with infection prevention, he conceptualized “contact infection,” that is, infection by germs that did not come from the environment, but came from other people through direct contact or close proximity. However, he would go on to conflate close proximity with the actual mechanism of transmission, engendering a confusion that would muddy understanding for decades.

Chapin believed that contact infection was the main mode of transmission of many diseases. But like any new theory, his encountered resistance: “I have sometimes been told I lay too much emphasis on contact infection,” he wrote, although “until recently very little attention has been paid to it.” He was no doubt aware of the resistance faced by Semmelweis, Snow, Pasteur, Koch, King, and many others, and realized the need to make his case forcefully if he was to convince his colleagues of the importance of contact infection.

Chapin also reviewed the possibility of airborne infection, which he conceived especially as infections from afar. He stated that “From time immemorial, and until a very recent period, the air has been considered the chief vehicle of infection,”^75^ but important diseases such as cholera, malaria, and childbed fever, that were for centuries thought to be transmitted through the air, had been shown to have other routes of transmission, and the belief about their airborne transmission had been shown to be erroneous. Nevertheless, airborne transmission was still considered so important for many diseases^76^ to warrant a response from Chapin, and the miasmatic ideas of phantasmagorical disease transmission through the air were still in the public's mind. As Chapin admitted at the end of that chapter, the lingering belief in airborne infection was the main obstacle he encountered to promote his ideas of the importance of contact infection. Echoing earlier concerns from Cornet,^70^ he stated “If the sick‐room is filled with floating contagium, of what use it is to make much of an effort to guard against contact infection? […] It is impossible, as I know from experience, to teach people to avoid contact infection while they are firmly convinced that the air is the chief vehicle of infection.”

Chapin was aware of the work of Flügge and at the UK House of Commons showing transport of germs for considerable distances and floating in the air for hours. He also did realize that airborne infection may explain infection in close proximity. However, he argued that ease of infection in close proximity was better explained by “spray‐borne” droplets, the large visible droplets considered by Cornet and others. He argued that since germs began to die or lose their virulence outside of the body, the closer we were to others, the greater the chance of infection. There were many opportunities for “transfer of secretions” between people during close contact. Infection from asymptomatic cases had been identified by Koch for cholera,^77^ or as in the famous instance of “Typhoid Mary,” an asymptomatic cook who infected 53 people with typhoid fever in New York City in 1907.^78^ Chapin used transmission from asymptomatic carriers as an argument to help dismiss the more apparently unexplainable transmission events, that had often been attributed to airborne transmission since the time of Hippocrates: “Now that the number of unknown foci of infection and the opportunities for direct transfer of secretions have been demonstrated, the deduction is certainly permissible that contact infection is more important [than other modes].”^74^

Chapin stated that “[t]here is no evidence that [airborne transmission] is an appreciable factor in the maintenance of most of our common contagious diseases.” And critically, he turned (an already not completely correct claim of) absence of evidence into evidence of absence. “We are warranted then, in discarding [airborne transmission] as a working hypothesis, and devoting our chief attention to the prevention of contact infection,” he concluded. “It will be a great relief to most persons to be freed from the specter of infected air, a specter which has pursued the race from the time of Hippocrates.” He later summarized his conclusions in a review in the prominent J. Am. Med. Assoc., stating that “There is little evidence that, among the diseases which commonly occupy our attention in this part of the world, aerial transmission is a factor of importance. […] We may be sure that the sewer gas bogey is laid, the notion that dust is a dangerous vehicle of every‐day infection is unsupported and that mouth spray is usually effective only at short distances.” He only left open the possibility for tuberculosis, although “the last word has not been said.”^75^

Neither Snow nor Semmelweis were highly recognized in public health before their major discoveries and, as is often the case, faced more resistance to their ideas.^79^ Chapin was much better positioned to change the paradigm of transmission, as the long‐serving Health Officer of Providence and also thanks to the success of his emphasis on contact transmission in reducing infections in a new hospital. In 1927, he became the President of the American Public Health Association. His ideas about the dominance of contact infection and the implausibility of airborne infection were incorrectly defined, as we will later examine, but were widely adopted in the fields of public health and infectious diseases. Chapin was described in 1967 as “the greatest American epidemiologist” by Alexander Langmuir, the first and long‐time director (1949–1969) of the epidemiology branch of the CDC, and as late as the 1980s, Chapin's views were dominant there.^80^ Critically, Chapin's unproven hypothesis was accepted as true: Ease of infection in close proximity is accepted proof of transmission from sprayed droplets. This key error conditioned the evolution of this field over the next century. Chapin's ideas were still dominant at the start of the COVID‐19 pandemic.

### 3.5 No important natural disease is airborne (1910–1962)

Influenza, thought in the 15th century to be caused by the noxious influence of winter constellations (“influenza delle stelle”), can cause severe pandemics when a significantly different strain emerges through genetic evolution. The most severe pandemic by far in the 20th century was that of 1918 (“Spanish Flu”). In the early stages of the pandemic, a warning from the US Surgeon General published in newspapers across the United States warned of “germs being carried with the air along with the very small droplets of mucus, expelled by coughing or sneezing, forceful talking, and the like.”^81^ The dangers of infection thus justified public health recommendations for the public to cover their coughs, avoid crowds, and wear masks when in the same room as infected persons. There was some evidence that ventilation and outdoor air reduced transmission, which suggested airborne transmission. For example, some cities such as Chicago implemented public health measures strongly focused on ventilation, including in schools, churches, and rooms where patients were being treated; places of public gathering, such as dance‐halls and theaters, were closed until thorough renovation works were carried out as a condition for a permit to reopen. Chicago had been the first city to adopt ventilation ordinances in public buildings and conveyances (including street cars) and in workplaces in 1910. The city reopened within 6 weeks and did not have a second wave of pandemic,^82^ although it may have fared better than other cities for a combination of reasons. However, the limited nature of the understanding of pathogen transmission that emerged during the pandemic was not enough to force a paradigm shift, and Chapin's ideas became firmly established over the next two decades.

In the 1930s, Harvard engineering professor William Wells and physician Mildred Wells, his wife, started applying more contemporary experimental methods to the investigation of airborne transmission. Chapin had successfully shifted the paradigm and his theory was now viewed as scientific progress, while the Wellses were accused of a retrograde approach to science which sought to bring back the miasma theory.^83^

William Wells was the first person to rigorously study the size of spray‐borne droplets vs. airborne aerosols. He conceptualized a dichotomy of spray‐borne droplets (≳100 μm) that reach the ground before they dry, vs. aerosols (≲100 μm) that dry before they reach the ground (thus referred to as “droplet nuclei”). He correctly understood the connection with meteorology where these facts are common knowledge,^84^ stating “A raindrop 2 mm in diameter can fall miles without completely evaporating under conditions which would cause a 0.2 mm droplet to evaporate before it had fallen from the height of a man.”^85^

The Wellses suspected that tuberculosis and measles were airborne, but both were already believed to be droplet diseases, and they encountered intense resistance from the epidemiological community. Measles was described by most public health institutions as a droplet disease as late as 1985, because of ease of transmission in close proximity and cases of lack of infection with shared air.^86^ The Wellses had some initial success showing that UV light installed in the upper zone of a room above the head height of occupants, such that only aerosols rising through thermal plumes would be exposed to UV, greatly reduced measles and chickenpox infection.^87^, ^88^ However, subsequent attempts to replicate these findings produced mixed results. In retrospect, the reason became apparent. In the schools where UV prevented transmission, children were together indoors only in the school, not elsewhere. Thus, disinfecting the school air was effective. In subsequent studies at other schools, the children shared other indoor spaces (such as school buses), for hours. Thus, there were plenty of opportunities for transmission of measles via shared indoor air that was not subject to UV disinfection. In a 1945 article in a predecessor journal to *Science*, W. Wells lamented how our societies had invested and been successful in eliminating infections through drinking water and food, but no action had been taken to limit airborne infection, since it was widely accepted that natural diseases were not airborne.^89^

**FIGURE 1:**
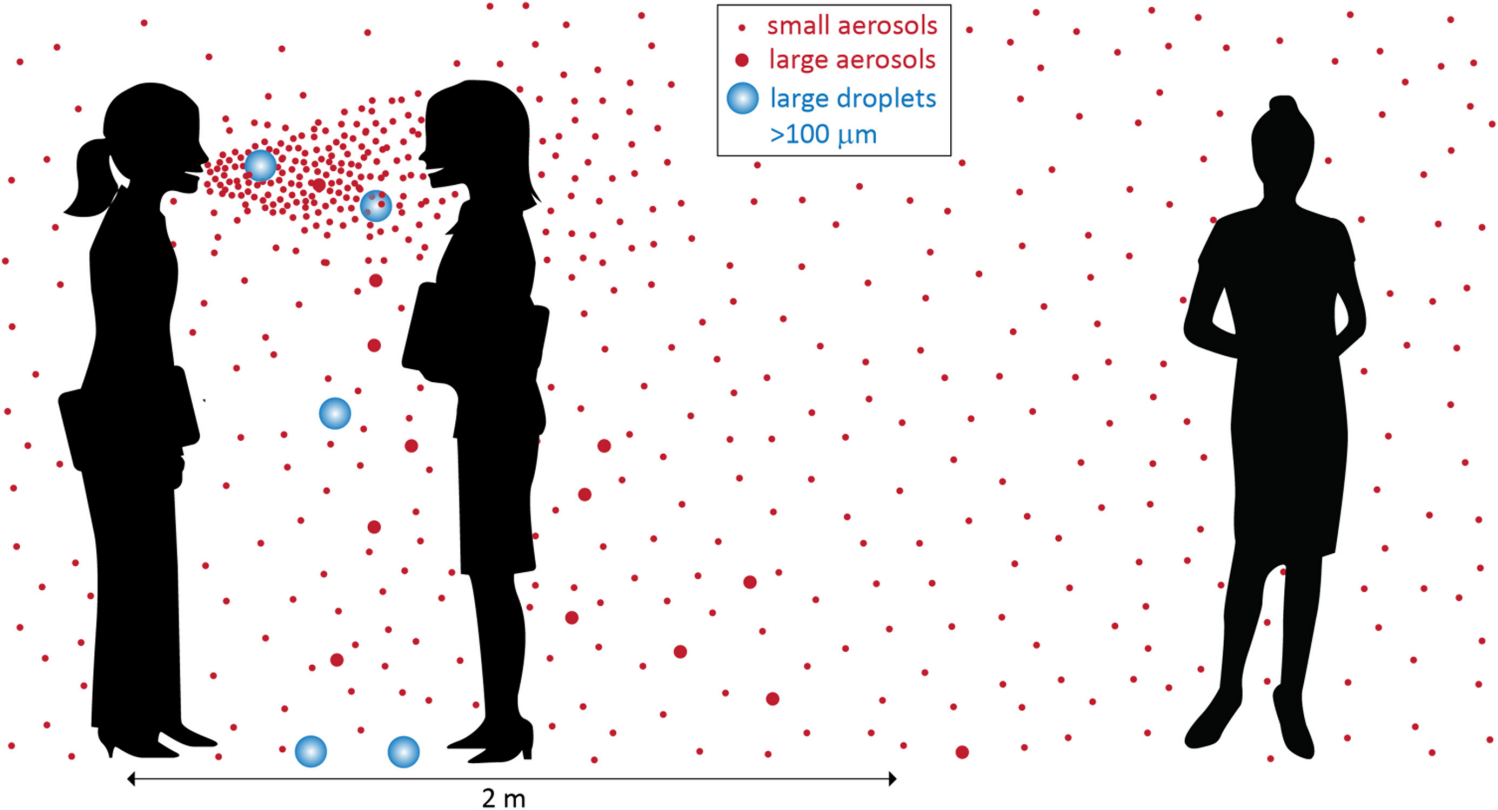
Illustration of droplets and aerosols released during talking; these may carry viruses if the person is infected. The large droplets fall rapidly to the ground in close proximity. The small aerosols are much more concentrated in close proximity, and they can remain floating in the air and spread throughout the room, leading to (reduced) exposure at a distance. Adapted from Tang et al^91^

In 1951, Langmuir stated, “It remains to be proved that airborne infection is an important mode of spread of naturally occurring disease.”^90^ Langmuir had worked on preventing infectious disease transmission among US military personnel during World War II. Substantial resources were dedicated to the effort, given the impact of disease outbreaks on military readiness, generating knowledge “which would have taken decades to accumulate under peacetime conditions” and that established the professional leaders in this area for the next several decades.^80^ However, Langmuir and collaborators had a key problem when trying to investigate airborne infection: they viewed the world through the lens of Chapin's theories. For example, in one study, crowding was reduced in military barracks in order to determine whether rates of illness decreased, with the reasoning that increasing distance would reduce close proximity (and thus prevent droplet‐based transmission). Conversely, if transmission were airborne, Langmuir expected that reducing crowding should have no impact. Reducing crowding reduced disease, thus “substantiating the role of droplet spread.”^80^ But the inference ruling out airborne infection was defective since it was ignoring the fact that the exhalation of an infected person is most concentrated in close proximity, with much dilution upon mixing with room air,^17^, ^27^, ^91^ as shown in Figure 1. The concept of gradual dilution of the exhaled aerosols with distance from the infector was somehow missing from their interpretation. The impact of Chapin's views was profound, leading to the misinterpretation of transmission studies over a century, including in dominant public health institutions such as the CDC.

However, Langmuir's work renewed interest in the physics of airborne infection, as he concluded that weapons of airborne disease can be created, which became a topic of intense interest during the cold war.^80^ Based on studies of occupational exposure, he learned that aerosols smaller than 5 microns can penetrate deeply into the lung, all the way into the alveolar region. Infectious disease aerobiology was extensively developed during this period as part of the US and Soviet Union bioweapons programs.^92^ However, most of the work remained classified even after the weapons were banned, and thus that body of work had little influence on the general medical and infection control communities. This may have contributed to the continued dominance of Chapin's paradigm.

### 3.6 Reluctant acceptance of as little airborne transmission as possible (1962–2020)

Despite the stubborn resistance to the idea that airborne transmission had any relevance for natural diseases, W. Wells, Robert Riley, and Cretyl Mills succeeded in demonstrating airborne transmission of tuberculosis (TB) in 1962 through extensive efforts. They routed the air from a tuberculosis ward to 150 guinea pigs for 2 years. About three guinea pigs per month were infected. However, none were infected in a control group where the only difference was that the air was irradiated with germicidal ultraviolet light, killing the TB bacterium.^93^, ^94^ Because of this study, TB was the first important natural disease to be accepted as airborne in modern times.

As this example shows, the standards of evidence were clearly different for different routes of transmission, as many diseases were accepted as “droplet” without any substantive proof—let alone such extensive and time‐consuming experiments. The resistance to a larger role for airborne infection continued, with a pattern of accepting airborne transmission on a case‐by‐case basis for each disease only when the evidence was undeniable—that is, only when all other transmission routes could be ruled out and the evidence was very clear.

For example, there was an obvious case of long‐distance airborne transmission of smallpox in Germany in 1970. A report on the outbreak reflected the ongoing thinking, concluding, after ruling out all other plausible infection routes: “The only remaining route of transmission considered reasonable was airborne spread of a virus‐containing aerosol, *a possibility against which all of the investigators were initially prejudiced*”^27^ (emphasis ours)^98^. In addition, the acceptance of airborne transmission was applied mainly to this outbreak, which was described as an unusual event, “a unique exception.”^80^ Droplet transmission continued to be considered dominant for smallpox. The success of the program to eradicate smallpox was taken as vindication of this view.^80^ However, when the actual biophysics of aerosols is correctly taken into account, the ease of infection in close proximity together with some cases of distant infection in shared indoor air with low ventilation is a signature of airborne transmission,^21^, ^27^ and there is evidence that airborne transmission of smallpox was more important than has been accepted so far.^96^ In addition, the smallpox incubation period was very precise: virtually 100% of infectious people were symptomatic, and viral shedding and transmission did not occur during the incubation period, but only when patients became symptomatic, at which time they were very sick and did not move around very much. Thus, the track/trace/isolate/quarantine/ring vaccination approach of the eradication program worked well, despite the potential for airborne transmission.^96^, ^97^

The same pattern of scientific inquiry played out for measles and chickenpox, two extremely contagious diseases, whose airborne character was resisted for seven decades and only finally widely accepted in the 1980s based on multiple superspreading events with long‐distance transmission (when the infector and infected were never together in the same room).^86^, ^98^ Importantly, ease of transmission in close proximity was observed for all accepted airborne diseases (hence their original classification as droplet diseases).^86^, ^99^, ^100^ But despite this overlap, ease of transmission in close proximity continued to be taken as evidence of droplet‐only transmission for other diseases. Lack of measles transmission with shared indoor air in some cases was also used as an argument against its airborne transmission. The same feature has been observed for COVID‐19, and is now understood to be due to very high variability in viral load and aerosol shedding among individuals, as well as differences in respiratory intensity and vocalization between different situations.^27^, ^101^, ^102^, ^103^, ^104^, ^105^, ^106^

The SARS‐CoV‐1 epidemics of 2003 brought renewed attention to the issue of airborne transmission. Superspreading was clearly observed.^107^ Airborne spread was implicated in several outbreaks in hospitals^108^, ^109^ and also in the large Amoy Gardens outbreak in Hong Kong, both through a building air shaft and possibly by outdoor plumes between the closely packed tall apartment buildings.^110^ However, the airborne designation of SARS‐CoV‐1 was not widely accepted in the infection control world.^111^ Although WHO describes SARS‐CoV‐1 as an airborne virus,^112^ a prominent member of the WHO COVID‐19 IPC Committee concluded in 2015 that “There is now general consensus that SARS is not airborne.”^113^ Part of the confusion arises from a too narrow use of the word “airborne” in which short‐range airborne transmission is interpreted as *only* droplet transmission, and only longer‐range airborne transmission is considered really airborne. After the 2003 SARS‐CoV‐1 outbreaks, intense concern was focused on the impact of “aerosol‐generating procedures” (AGPs). These are medical procedures such as bronchoscopy, intubation, and suctioning, which were thought to generate large amounts of aerosols and to have infected some of the medical staff performing them during the SARS‐COV‐1 outbreaks, although the evidence supporting this association was weak.^114^, ^115^ This line of reasoning also ignores the fact that although AGP may lead to the release of aerosolized viruses as shown, for example, with influenza A,^116^ so will other non AGP activities such as coughing or breathing which can lead to a sizeable aerosol dose in the vicinity of an infected patient.^116^, ^117^

During the last several decades and until the COVID‐19 pandemic, with available antibiotics, vaccines, and no major respiratory pandemics, studies further probing the details of droplet vs. airborne transmission had not been a major public health priority. The aftermath of the Oil Crisis and then the Climate Crisis have led to compromises in building standards in favor of energy saving over ventilation and public health.^118^ The high standards of ventilation and filtration adopted in many clinical spaces in modern hospitals^119^, ^120^, ^121^ mean that airborne risks have been substantially mitigated in these settings, where many key infection control scientists work. However, this is not the case in all hospital spaces or for older hospitals dependent upon natural ventilation. Adherents of droplet transmission were in control of all key public health institutions, and scientists proposing airborne transmission were typically ignored.^69^

Evidence also points to the importance of airborne transmission for another disease with high pandemic potential: influenza,^122^, ^123^, ^124^ including superspreading in poorly ventilated indoor air,^125^, ^126^ low transmission in well‐ventilated environments,^127^ exhaled infectious virus^128^, ^129^ and viral^117^ detection (of both infectious virus and viral RNA) in room air,^130^, ^131^, ^132^ 100 times smaller dose by inhalation of aerosols vs. intranasal inoculation,^133^, ^134^, ^135^, ^136^ and airborne transmission in animal models.^137^, ^138^ However, likely due to the same kinds of resistance as described above for other diseases, airborne transmission of influenza virus has not been widely accepted, and it is still described by WHO and CDC on their websites as a droplet/fomite disease, with no mention of airborne transmission.^139^, ^140^

There is also evidence for airborne transmission of rhinovirus,^141^, ^142^, ^143^, ^144^, ^145^ adenovirus,^146^ SARS‐CoV‐1,^110^, ^147^ MERS‐CoV,^148^, ^149^ and RSV.^150^, ^151^ Limited data suggest a role of airborne transmission for enteroviruses,^152^, ^153^ filovirus,^154^ and other pathogens.^155^

Furthermore, airborne transmission of viruses is well accepted in veterinary medicine including for some coronaviruses and influenza viruses, sometimes over distances of many kilometers. Examples include the foot and mouth virus,^156^, ^157^ porcine reproductive and respiratory syndrome virus (PRRSV),^158^, ^159^ porcine respiratory coronavirus,^160^ avian infectious bronchitis virus (also a coronavirus),^161^ and equine influenza.^162^, ^163^

### 3.7 The COVID‐19 pandemic and the uncovering of the historical error

Just as the COVID‐19 pandemic was getting started, Chen et al.^17^ reported that “Reviewing the literature on large droplet transmission, one can find no direct evidence for large droplets as the route of transmission of any disease.”.^17^ One of the earliest reports about the early outbreaks in China in the prominent Nature journal concluded that “the disease could be transmitted by airborne transmission, although we cannot rule out other possible routes of transmission.”^7^ Some early public health announcements in China reported that the novel coronavirus was airborne.^8^

However, and despite a lack of direct evidence in favor of droplet or fomite transmission, by March 2020 public health institutions like WHO concluded that ease of transmission in close proximity proved that COVID‐19 was transmitted by those mechanisms,^3^ continuing Chapin's 1910 error. Key experts from the WHO IPC committee implied that they would recognize an airborne disease given an expected high R_0_,^32^ despite a delay of 70 years to recognize measles and chickenpox as airborne,^86^, ^98^ and despite the fact that pulmonary tuberculosis is exclusively airborne and yet less contagious than COVID‐19.^164^ Interestingly, despite publications with the types of evidence that were sufficient for accepting tuberculosis (animal experiments^165^), and measles/chickenpox (superspreading and long‐distance transmission, e.g. Ref.^166^, ^167^, ^168^) as airborne, WHO and other public health agencies continued to resist the importance of airborne transmission of COVID‐19 for almost a year. The public health establishment remained entrenched in the old droplet paradigm. It considered the evidence of airborne transmission provided by the aerosol scientists, who were rebuffed and excluded from key committees, as weak or irrelevant.^13^, ^31^ The same pattern discussed above, that is, minimizing the role of airborne transmission as much as possible, was on display, through the use of terms like “situational airborne,” or by claiming airborne transmission is restricted only to poorly ventilated crowded locations. This is an error in logic, since all airborne pathogens are very sensitive to ventilation, e.g. Ref.^169^ and if they can infect in shared room air, they must be much more infective in close proximity where they are much more concentrated (Figure 1).^91^ Thus, if a pathogen is airborne in poorly ventilated locations, respirators should also be worn to protect from it in close proximity.

Over the course of a year, accumulating evidence that COVID‐19 is a predominantly airborne disease made clear that it was a logical error to conflate infection in close proximity exclusively with droplet transmission.^21^, ^91^, ^170^ Lack of control of the pandemic through only droplet/fomite measures such as physical distance, handwashing, and surface disinfection became apparent, as did multiple cases of unambiguous long‐range airborne transmission such as in quarantine hotels.^167^, ^168^, ^171^, ^172^, ^173^ Cases of transmission in hospitals despite surgical masks and eye protection^174^, ^175^ and between patients sharing a room despite distance and physical barriers were also published.^176^ WHO^12^ and CDC^16^ finally partially accepted airborne transmission of SARS‐CoV‐2 in April/May 2021 as important. However, the changes as of January 2022 were often expressed confusingly and had received insufficient publicity,^20^ and changes in the mitigation measures were only partially reaching most of the world. Some of the emerging SARS‐CoV‐2 variants‐of‐concern were more transmissible,^23^, ^177^ and for this reason, the cases of airborne superspreading or long‐distance transmission have become easier to identify.

It has also become clear that some public health organizations would at times use the concept of ‘short‐range’ or ‘close‐contact’ transmission via “droplets” as due to particles that can be inhaled, which is actually describing an aerosol phenomenon. To be inhalable, particles need to be smaller than about 100 μm.^178^ They are thus aerosols that can travel beyond close proximity of the infected person.^85^, ^179^ Milton^1^ proposed avoiding the potentially ambiguous term “droplet,” and using the terms “aerosols” for smaller particles that can be inhaled, and “drops” for the larger particles that fall to the ground, being too heavy to be inhaled. Li proposed referring to the mechanisms as aerosol inhalation, surface touch, and drop spray,^2^ and those definitions were adapted by the CDC in 2021.^16^

WHO commissioned in 2020 a series of systematic reviews on the transmission of SARS‐CoV‐2 to a specific group. WHO commissioned a systematic review on airborne transmission with no aerosol science input, despite the cross‐disciplinary complexity of the topic. Airborne transmission was reviewed in a very narrow way, only considering one type of evidence, namely the detection of viable virus in air,^180^ despite the fact that this has not been achieved for accepted airborne diseases such as tuberculosis, measles, and chickenpox.^4^, ^181^ The many other types of evidence that support airborne transmission as predominant for SARS‐CoV‐2 and that led to acceptance of tuberculosis, measles, and chickenpox as airborne^21^, ^86^, ^87^, ^93^, ^98^ were ignored in the review. As of this writing, the paper had not passed peer‐review, and the public comments from other scientists remained unanswered. e.g. Ref.^182^ A review was written for “close contact,”^183^, ^184^ which appears to be a conceptual error since close contact is a measurement of distance* F and not a mechanism of transmission. No review has been posted summarizing the evidence supporting droplet transmission, despite WHO and key coauthors stating that it is the main mechanism of transmission.

AGPs were the only circumstance in which WHO clearly accepted airborne transmission as of mid‐2020.^10^ However, multiple studies during the COVID‐19 pandemic showed that patients produce more aerosols through simply breathing, talking, singing, and coughing than from many AGPs.^185^, ^186^, ^187^, ^188^, ^189^ Although the initial precaution was probably warranted, the continued emphasis on AGPs as a much higher airborne transmission risk than from naturally produced aerosols was misguided, but had not been widely corrected as of this writing.

**FIGURE 2:**
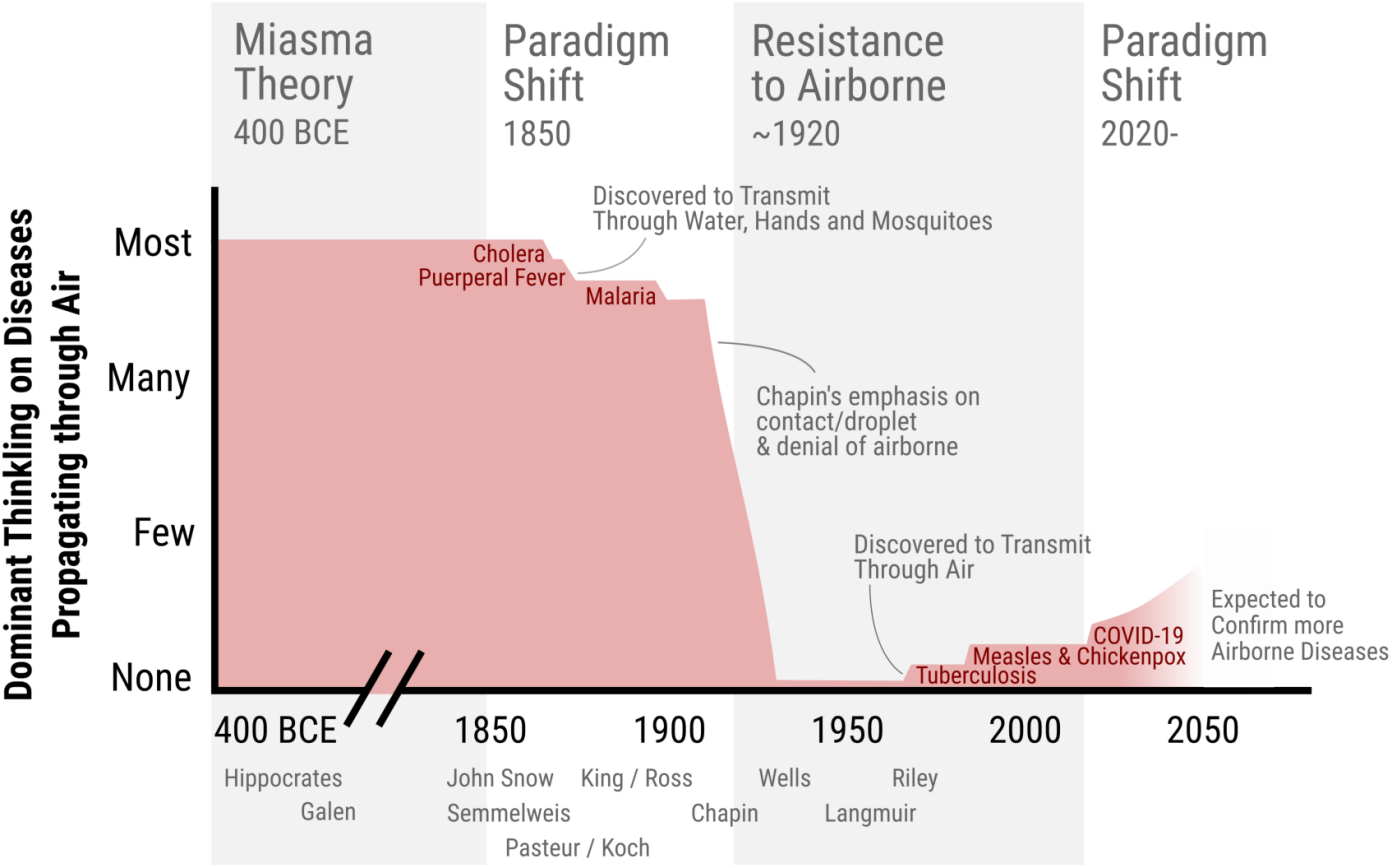
Qualitative representation of the dominant medical/public health thinking in the West about how many important diseases transmit through the air, with some critical steps and practitioners marked as text

Figure 2 qualitatively illustrates the shift in dominant paradigms over time about disease transmission through the air. The miasmatic paradigm/dogma, in which foul air led to disease, prevailed for two millenia. This paradigm was weakened by the discovery that multiple diseases (e.g., cholera, puerperal fever, and malaria) that had been thought to transmit through the air, were in reality transmitted by a variety of other means, and by the acceptance of germ theory. Then, in around 1912, Chapin wrote Sources and Modes of Transmission, a book that cataloged disease transmission modes. He noted that germs lived in the body but not well outside, thus incorporating germ theory into study of disease transmission, and posited that most infection was transmitted by contact, meaning touch or short‐range transmission, which to him was explained by spray‐borne droplets. The success of his theories overturned the previous paradigm, and led to the opposing paradigm/dogma of droplet transmission for all respiratory diseases, with airborne transmission thought to be unimportant for disease transmission by the 1930s. The second half of the 20th century saw very limited acceptance of a few diseases as airborne, amidst great resistance.^190^ The COVID‐19 pandemic brought enormous scrutiny on the subject and made the errors inherent in the droplet dogma well known, hopefully ushering a more objective paradigm for airborne transmission.

### 3.8 The lessons from the persistence of the 5 micron error

During the COVID‐19 pandemic, a different error also became apparent. Public Health documents such as the July 2020 WHO Scientific Brief on COVID‐19 transmission^10^ (still the latest WHO brief on the topic as of this writing) repeat a long‐standing error in previous guidance and scientific literature: They place the separation between droplets that fall to the ground in 1–2 m and aerosols that remain airborne at 5 microns. The correct value is of the order of 100 μm, with an estimated range of 60–100 μm depending on the specific conditions^179^ (an error of a factor of over a thousand in the mass of the particles). Aerosols smaller than ~30 μm can stay aloft more than one minute, while those in the nominal range 30–100 μm will deposit faster and will generally be inhaled in close proximity and deposit exclusively in the upper respiratory tract.^191^ Particles larger than about 100 μm cannot be inhaled^192^ and can only infect by the spray‐borne droplet impact mechanism. When talking, droplets need to be larger than about 300 μm to be able to impact onto another person at conversational distances of >0.6 m, as smaller droplets do not have enough inertia to cross the air gap and reach the other person.^17^, ^193^ The correct boundary was published by Wells in 1934,^85^ and is shown in the CDC webpage^194^ (occupational medicine branch). It has been confirmed by more recent publications from aerosol scientists,^179^ and again multiple times during the COVID‐19 pandemic, including a workshop of the US National Academies of Science, Engineering, and Medicine.^170^, ^195^ However, the error has persisted in the scientific literature and guidance documents and was not corrected by WHO as of March 2022. Randall et al.^13^, ^69^ have investigated the source of this error, and traced it to the 1960s, where tuberculosis was the only accepted airborne infection, which appears to have led to a confusion between the particle size that penetrates the deep lung (necessary for TB infection) and that falls to the ground in 1–2 m.

The fact that the 5 micron error was able to persist for so long, and is still present in WHO's latest scientific brief on transmission of a major pandemic virus, is puzzling. In our opinion, it is a consequence of the overwhelming dominance of Chapin's paradigm in infection prevention and epidemiology, where droplet infection is the assumed mode of transmission of respiratory diseases unless proven extremely conclusively otherwise. This dominance led to a persistent lack of attention to the details of the physics of airborne transmission, and to the input from disciplines such as aerosol science and even occupational medicine.

Because aerosols (up to 100 μm) can follow air currents, the recognition of their complete size spectrum is important for the selection of PPE that will provide a seal around the airways (e.g., N95/FFP2). Also, wider recognition that only small‐size aerosols can penetrate into the lower respiratory tract (<20 and <5 μm for the alveolar space)^178^ has important implications for infections affecting only the lower respiratory tract, such as tuberculosis or legionellosis. Another relevant example, given the topicality of emerging coronaviruses, might well be MERS‐CoV^111^ which has been shown to replicate preferentially and extensively in the lower respiratory tract,^196^, ^197^ with high viral load detected in clinical samples from the lower respiratory tract (LRT). In contrast, samples from the upper respiratory tract (URT) show a much lower, sometimes undetectable, viral load.^198^, ^199^, ^200^ The lack of detectable MERS‐CoV subgenomic RNAs from nasopharyngeal swabs,^201^ reports of the failure to detect expression of the DPP4 receptor in URT epithelium,^202^, ^203^ (although dissenting data exist^204^) and the failure to detect expression of an alternate receptor^203^ suggest that URT replication may not occur at all. In turn, all this evidence points to an important, perhaps necessary, role for transmission by small‐size aerosols. This is supported by recovery of infectious MERS‐CoV in air samples from patient wards^148^ and successful experimental infection of rhesus macaques and African green monkeys using aerosol inocula.^149^

## 4 OUTLOOK FOR CONTROL OF RESPIRATORY DISEASES AND THE NEXT PANDEMIC

This overview of the history illustrates the pervasiveness of “belief perseverance,” the psychological tendency to maintain a belief despite clear and strong new evidence that should challenge it, especially in the context of institutional incentives that favor inertia and resistance to change.^36^, ^37^, ^205^ In an era of amazing scientific advances, with very rapid vaccine development following virus sequencing obtained in a few days, the very slow acceptance of critical new knowledge reminds us that the human aspects of science remain as pervasive as they were in past eras.

The persistence of the droplet paradigm may have been aided by several other reasons. First, even if the mechanism is incorrect, it still works reasonably well to reduce infection from airborne diseases, especially less contagious ones that mostly transmit in close proximity.^27^, ^206^ Distance from an infectious person will always increase the dilution of exhaled air and reduce such transmission.^27^, ^206^ Unfortunately, major systematic problems arise when a true empirical fact (distance reduces transmission) is used to reach the incorrect conclusion (the mechanism is spray‐borne droplets), and then, the incorrect mechanism is used to deduce what other measures may be protective. For example, during the COVID‐19 pandemic billions of dollars were spent putting up lateral plexiglass barriers in schools to block droplet projectiles (even though such barriers have actually been shown to increase SARS‐CoV‐2 transmission^29^) rather than opening the windows or wearing masks. Second, spray‐borne droplets are relatively easy to protect against, just keep your distance and wash hands and you should be quite safe. It thus provides simple rules to communicate to healthcare workers and the general population. Third, it removes the intense fear that airborne transmission can cause, and that has been associated with it throughout history. The historical fear often appears to be rooted in the more phantasmagorical conception of airborne transmission: The infected air can reach a person anywhere, and there is little that one could do to protect oneself from it. Critically, the logic leading to the fear did not account for the importance of dilution, and the feasibility of using it to reduce transmission. The irrational fear caused by this lack of understanding is paralyzing and creates real‐world problems for controlling disease transmission, as summarized, for example, in the above quotes from Cornet and Chapin: Either people just gave up, or extreme measures were needed such as treating tuberculosis patients like lepers.^70^, ^74^ Fourth, given that strict airborne transmission prevention measures can be costly or unavailable at large scale in healthcare facilities (e.g., negative pressure rooms in hospitals), there was a reluctance of public health organizations to declare a widespread virus such as SARS‐CoV‐2 during the pandemic as airborne, out of fear of the budgetary, legal, and labor consequences. Governments also seemed content to promote measures that only require personal responsibility, such as handwashing, and were much more reluctant to explain airborne transmission clearly as it would require costly actions on their part, for example, to improve ventilation and filtration in public buildings. Finally, a desire to save face by some authorities may have also played a role. They had emphatically declared airborne transmission of SARS‐CoV‐2 to be “misinformation,” and it could be embarrassing to subsequently acknowledge the importance of airborne transmission, which may perhaps qualify as one of the largest errors in the history of public health. In the private words of a public health advisor to a national government, “an approach is needed that will allow [us] to save face.”

Thankfully, the intense research and debate associated with the COVID‐19 pandemic have finally begun to generate a new paradigm shift in the understanding of disease transmission. Not only are respiratory diseases not transmitted exclusively by droplets, but also it is likely that many or most respiratory diseases have an important, if not predominant, airborne component of transmission.^191^ It is also clearer that for a respiratory disease to have pandemic potential, airborne transmission is likely to be an essential component. This does not mark a return to past miasmatic ideas, but a more informed understanding of airborne transmission as more complex and less scary than in the past, and certainly as a tractable problem.^33^, ^207^, ^208^ This new paradigm has major implications for the regulation and control of air quality in indoor spaces, by proper ventilation, filtration, and other means, as well as for PPE for workers and masking by the public. Finally, the lack of attention to the quality of shared indoor air that Wells lamented in 1945^89^ may finally start to be remedied in the coming years,^33^ potentially leading to a reduction in respiratory disease transmission for decades to come.

## AUTHOR CONTRIBUTIONS

J.L. Jimenez: Conceptualization; Investigation (lead); Writing – Original Draft. Other authors: Investigation (equal); Writing – review and editing (equal).

## CONFLICT OF INTEREST

The authors declare no conflict of interest.

## Data Availability

The main data sources for this literature review are those listed in the literature reference list.

## Endnote

* F Strictly speaking “close contact” includes metrics of distance and time, e.g. <6 feet for more than 15 min in the US CDC definition.
